# Changes in Renal Function After Nephroureterectomy for Upper Tract Urothelial Cancer in Elderly Patients

**DOI:** 10.7759/cureus.61479

**Published:** 2024-06-01

**Authors:** Tatsuya Kawamura, Daiki Ikarashi, Arisa Machida, Daichi Tamura, Tomohiko Matsuura, Shigekatsu Maekawa, Renpei Kato, Mitsugu Kanehira, Ryo Takata, Wataru Obara

**Affiliations:** 1 Urology, Iwate Medical University, Yahaba-cho, JPN; 2 Hospital Medicine, Iwate Medical University, Yahaba-cho, JPN

**Keywords:** hydronephrosis, elderly, renal function, nephroureterectomy, upper tract urothelial carcinoma

## Abstract

Introduction

Decreased renal function after radical nephroureterectomy is one of the most important complications because it contributes to the decision to initiate adjuvant chemotherapy. This study aimed to investigate clinical factors associated with changes in renal function after radical nephroureterectomy in elderly patients.

Methodology

A total of 145 patients who underwent radical nephroureterectomy for upper tract urothelial carcinoma were evaluated. The renal function was calculated preoperatively, postoperatively, and one month postoperatively, and the long-term change in renal function was investigated once a year. The association between clinical factors and changes in renal function following radical nephroureterectomy in univariate and multivariate analyses was stratified by age ≥75 years and <75 years.

Results

The median age of the patients was 71 years, with 94 patients (65%) aged <75 years and 51 patients (35%) aged ≥75 years. The median estimated glomerular filtration rates (eGFRs) were 57.1 (21.8-100) preoperatively, 36.1 (9.1-100) postoperatively, and 42.4 (19.5-100) in one month after radical nephroureterectomy. The median eGFRs in elderly patients were 50.8 (21.8-85.4) preoperatively. In the elderly group, only 8% had an eGFR of ≥50 as cisplatin-eligible at one month postoperatively. The long-term renal function in the elderly may decline further than during the stable postoperative periods. In the multivariate analysis, hydronephrosis (HN) was a significant predictor of decreased renal function in patients aged ≥75 years between the pre- and postoperative periods.

Conclusions

Elderly patients with HN who have upper tract urothelial carcinoma have a lower risk of decreased renal function after radical nephroureterectomy. This result may be useful in determining adjuvant therapy.

## Introduction

Upper tract urothelial carcinoma (UTUC) is a rare disease, occurring in 5% of all urothelial carcinomas. Due to fewer symptoms and delayed diagnosis, tumors are often locally advanced at onset, resulting in a lower survival rate than that of urothelial carcinoma of the bladder [1]. Although the standard treatment for localized UTUC has been radical nephroureterectomy (RNU), adjuvant therapy is optional to prevent recurrence and improve prognosis. A phase Ⅲ POUT trial showed a significant disease-free survival benefit with postoperative platinum-based regimen chemotherapy [2]. In this trial, cisplatin was used in approximately 60% of patients, depending on the eligibility for cisplatin use. Therefore, predicting renal function decline with RNU is important in using platinum-based regimens in adjuvant chemotherapy (AC). Most recently, immune checkpoint inhibitors, such as nivolumab, obtained FDA approval for treating urothelial carcinoma, including UTUC, in the adjuvant setting after RNU [3]. Therefore, our options in adjuvant therapy for UTUC have also expanded in patients with decreased renal function after RNU. Adjuvant therapy options have increased, but no clear criteria for appropriate treatment selection exist.

Especially, since UTUC has a peak incidence in elderly patients aged 70-90 years [4], the patient’s general condition, comorbidities, and renal function should be considered when deciding on adjuvant therapy. However, the consensus for adjuvant treatment for elderly patients with UTUC is still unclear. One of the major factors is the limited use of platinum drugs due to decreased renal function caused by RNU. Kaag et al. [5] reported that the median decrease in estimated glomerular filtration rate (eGFR) was 24% of the presurgical value. It is well known that kidney function declines with age, but few reports have examined changes in postoperative renal function in elderly patients with UTUC.

This study focused on elderly patients to evaluate changes in renal function after RNU and identify factors that predict renal function decline.

## Materials and methods

Between April 2012 and August 2022, 145 patients who underwent RNU for localized UTUC at Iwate Medical University Hospital were retrospectively evaluated. We divided the patients into two groups: the elderly group, those who were aged ≥75 years, and the nonelderly group, those who were aged <75 years. The indications for NAC were locally advanced high-risk UTUC, including cT3-4 and cN+ diseases. Moreover, the indications for AC were pT3-4 and pN+ diseases with platinum eligibility. We fundamentally used cisplatin-based regimens, such as gemcitabine plus cisplatin, for NAC and AC based on the eGFR following the EAU guideline [1]. Moreover, we used gemcitabine plus carboplatin (GCa) for cisplatin-unfit patients. We applied three courses of NAC and two courses of AC. Patients who received nivolumab as adjuvant therapy were excluded from the study. This study was conducted in accordance with the principles of the Declaration of Helsinki. The human ethics board of our institution approved this study, and written informed consent was obtained from all patients before enrollment (Iwate Medical University; protocol no. 2019-083).

The included variables were eGFR, age, sex, medical history, clinical/pathological T stage, preoperative hydronephrosis (HN), NAC, AC, and volume of the sound-side kidney corrected for height. The eGFR was calculated preoperatively when preoperative examination, postoperatively when the day after surgery, and one month postoperatively using the Modification of Diet in Renal Disease equation:$eGFR(mL/min/{1.73m}^2\ )=194\times{(serum\ creatinine)}^{-1.094}\times{(age)}^{-0.287}\times0.739(if\ female)$ [6]. This evaluated timing was selected as appropriate when the pathology results of the surgery were known, and the indication for adjuvant therapy was considered. The oncological outcomes of this study were overall survival (OS) and progression-free survival (PFS). OS was defined as the time from surgery to death or last follow-up, and PFS was defined as the time from surgery to radiographic or clinical progression or death.

We assessed the association of each clinical factor and changes in renal function following RNU in the univariate and multivariate analyses. The dependent variable was chronic kidney disease (CKD) stage change preoperatively and at one month postoperatively. The long-term changes in renal function were investigated once a year. Survival curves were plotted using the Kaplan-Meier method to describe OS and PFS. Statistical analyses were performed using JMP software (SAS Institute, Inc., Cary, NC). For all statistical comparisons, differences with a *P*-value < 0.05 were considered statistically significant.

## Results

Patient characteristics are described in Table 1. The median age at RNU was 71 years; 94 patients (65%) were aged <75 years, and 51 patients (35%) were aged ≥75 years. Moreover, 25 patients (17.2%) received NAC, and 22 patients (15.2%) received AC. There was no significant difference in clinicopathological factors, including medical history, between younger and elderly patients.

**Table 1:** Patients characteristics. eGFR, estimated glomerular filtration rate; RNU, radical nephroureterectomy

Valuables	All patients (*n* = 145)	Ages (years)		
		75 (*n *= 94)	≧75 (*n *= 51)	*P*-value
Renal pelvic cancer, *n* (%)	83 (57%)	52 (55%)	31 (61%)	0.74
Ureteral cancer, *n* (%)	62 (43%)	42 (45%)	20 (39%)	0.33
Male, *n* (%)	96 (66%)	59 (63%)	37 (73%)	0.75
Female, *n* (%)	49 (34%)	35 (32%)	14 (27%)	0.44
Tumor laterality right, *n* (%)	68 (47%)	45 (48%)	23 (45%)	0.28
Tumor laterality left, *n* (%)	77 (53%)	49 (52%)	28 (55%)	0.48
Clinical T1/T2, *n* (%)	108 (74%)	71 (76%)	37 ( 73%)	0.2
Clinical T3/T4, *n* (%)	26 (18%)	14 (15%)	12 (24%)	0.88
Clinical Tany, N＋, *n* (%)	11 (8%)	9 (10%)	2 (4%)	0.14
Pathological Tis/Ta/T1, *n* (%)	67 (46%)	53 (56%)	14 (27%)	0.31
Pathological T2, *n* (%)	17 (12%)	8 (9%)	9 (18%)	0.62
Pathological T3/4, *n* (%)	61 (42%)	33 (35%)	28 (55%)	0.54
Tumor low grade, *n* (%)	27 (19%)	20 (21%)	7 (14%)	0.25
Tumor high grade, *n* (%)	118 (81%)	74 (79%)	44 (86%)	0.76
Neoadjuvant chemotherapy, *n* (%)	25 (17%)	18 (19%)	7 (14%)	0.62
Adjuvant chemotherapy, *n* (%)	22 (15%)	14 (15%)	8 (16%)	0.29
Hydronephrosis, *n* (%)	60 (41%)	39 (41%)	21 (41%)	0.6
Hypertension, *n* (%)	67 (46%)	39 (41%)	28 (55%)	0.26
Hyperlipidemia, *n* (%)	32 (22%)	18 (19%)	14 (27%)	0.15
Diabetes mellitus, *n* (%)	27 (19%)	15 (16%)	12 (24%)	0.82
Median preoperative eGFR (mL/min/1.73 m^2^)	57 (21.8-100)	61 (27.6-100)	51 (21.8-87.2)	0.46
Median postoperative eGFR (mL/min/1.73 m^2^)	36 (9.1-100)	38 (15-100)	33 (9.1-69.3)	0.17
Median eGFR of one month after RNU (mL/min/1.73 m^2^)	42 (19.5-100)	44 (25.1-100)	37 (19.5-90)	0.06
Median volume of contralateral kidney (cm^3^/height (m))	87 (43-157)	89 (43-157)	83 (48-144)	0.21

**Figure 1:**
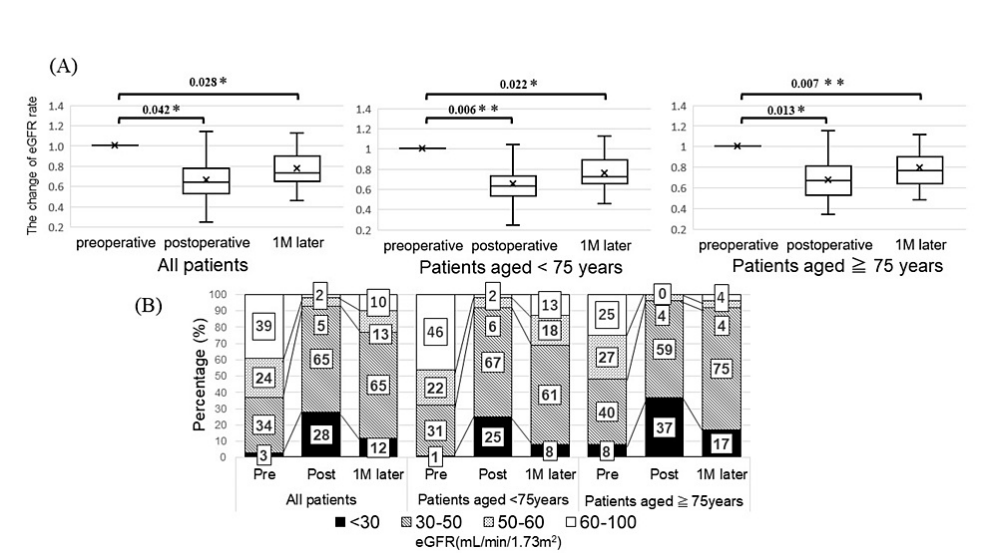
(A) The change ratio of eGFR for all patients, nonelderly patients, and elderly patients (calculated based on preoperative eGFR); (B) the percentage of patients by the eGFR preoperatively, postoperatively, and at one month after RNU in all patients, nonelderly patient, and elderly patients. ^*^P < 0.05. ^**^P < 0.01. RNU, radical nephroureterectomy; eGFR, estimated glomerular filtration rate

In the preoperative period, the mean eGFRs for all patients were 57.1 (21.8-100) for the nonelderly group 60.3 (27.6-100) and 50.8 (21.8-87.2) for the elderly group. The average eGFRs during the perioperative period is shown in Figure 1A. The average eGFR changes in all patients were −34.6% (0-75.4) between the preoperative and postoperative periods and 14.2% (−19.7-73.8) between the postoperative period and one month postoperative period. The average eGFR changes in the nonelderly group were −35.0% (0-75.4) between the preoperative and postoperative periods and 15.0% (−19.7-59.3) between the postoperative period and one month postoperative period. The average eGFR changes in the elderly group were −32.8% (2.1-66.1) between the preoperative and postoperative periods and 12.5% (−6.7-73.8) between the postoperative period and one month postoperative period. There was a significantly difference in the average eGFR changes between the preoperative and postoperative periods and postoperative period and one month postoperative period, and preoperative period and one month postoperative period in all groups (Figure 1A). Figure 1B shows in detail the eGFRs and patient proportions in the perioperative period. Patients who had an eGFR ≥ 50 at the preoperative period were 63% (59) of all patients, 68% of the nonelderly group, and 52% of the elderly group. At one month postoperatively, 31% of patients in the nonelderly group were cisplatin-eligible, while only 8% of the patients in the elderly group were cisplatin-eligible. Furthermore, 8% of patients in the nonelderly group were cisplatin-eligible, while only 8% of patients in the elderly group were cisplatin-eligible. Additionally, 8% of the nonelderly group and 17% of the elderly group had an eGFR < 30 at one month after RNU. ​​​​​ 

**Figure 2:**
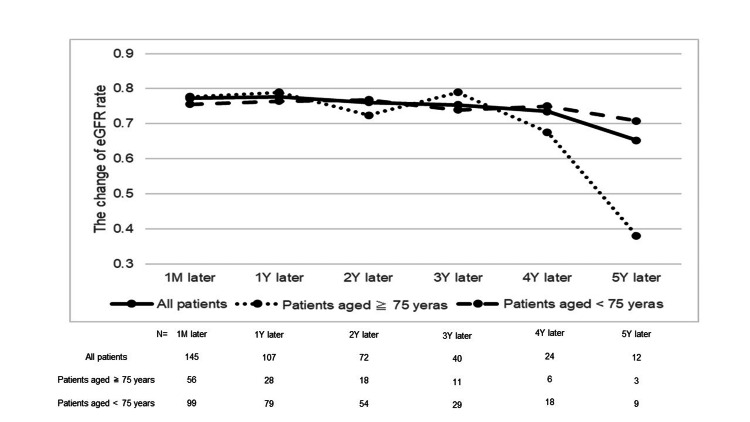
The long-term date of the change ratio of eGFR in all patients, nonelderly patient, and elderly patients (calculated based on preoperative eGFR). eGFR, estimated glomerular filtration rate

During the first five years postoperatively, there was no significant change in renal function in any patients. However, at five years postoperatively, the average eGFR change in elderly patients was -61.9%, compared to -34.7% in non-elderly patients (Figure 2). This showed that the long-term renal function in the elderly may decline further than during the stable postoperative periods.

In all patients, HN, ≥pT2, and the primary lesion were identified as factors associated with less decreased renal function between the preoperative period and one month postoperatively in the univariate analysis. HN and ≥pT2 were identified as factors associated with less decreased renal function in multivariate analysis (Table 2).

**Table 2:** Association between preoperative variables and renal function change from preoperative to one month after surgery. Hyphen (-)  means no date. ^*^P-value < 0.05 are considered statistically significant OR, odds ratio; CI, confidence interval

Variables	Univariate	Multivariate	
	*P*-value	OR (95% CI)	*P*-value
Age	0.65	-	-
Sex	0.76	-	-
Primary lesion	0.008^＊^	0.70 (0.25-1.93)	Neoadjuvant
Pathological stage	0.014^＊^	0.64 (0.48-0.91)	0.013^＊^
Tumor grade	0.12	-	-
Neoadjuvant chemotherapy	0.42	-	-
Hydronephrosis	0.021^＊^	0.98 (0.96-0.99)	0.029^＊^
Hypertension	0.73	-	-
Hyperlipidemia	0.73	-	-
Diabetes mellitus	0.34	-	-
The volume of contralateral kidney	0.56	-	-

In the nonelderly group, no clinical factors were related to decreased renal function. In the elderly group, only HN was identified as a factor associated with lower risk of decreased renal function in the multivariate analysis (Table 3).

**Table 3:** Association between preoperative variables and renal function change from preoperative to one month after surgery with patients aged ≧75 years. Hyphen (-)  means no date. ^*^P-values < 0.05 are considered statistically significant OR, odds ratio; CI, confidence interval

Variables	Univariate	Multivariate	
	*P*-value	OR (95% CI)	*P*-value
Sex	0.99	-	-
Primary lesion	0.2	-	-
Pathological stage	0.036^＊^	0.64 (0.33-1.2)	0.23
Tumor grade	0.09	-	-
Neo adjuvant chemotherapy	0.12	-	-
Hydronephrosis	0.044^＊^	0.91 (0.88-0.96)	0.04^＊^
Hypertension	0.97	-	-
Hyperlipidemia	0.37	-	-
Diabetes mellitus	0.66	-	-
The volume of contralateral kidney	0.15	-	-

**Figure 3:**
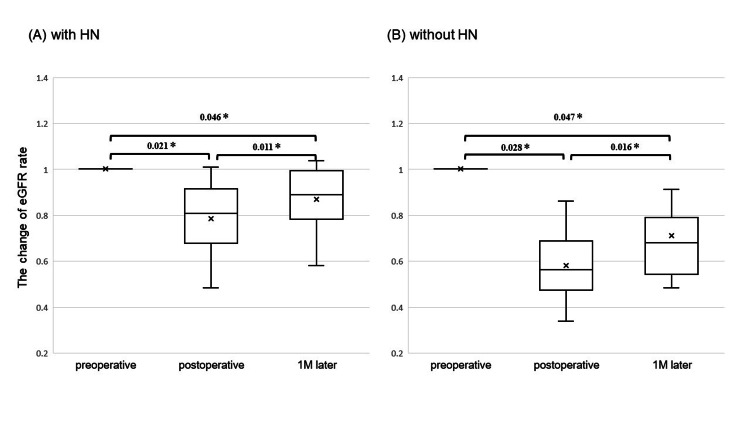
The change ratio of estimated glomerular filtration rate (eGFR) for elderly patients (A) with HN and (B) without HN (calculated based on preoperative eGFR). ^*^P < 0.05. HN, hydronephrosis; eGFR, estimated glomerular filtration rate

The average eGFR changes in elderly group with HN were −21.6% (0-51.7) between the preoperative and postoperative periods and 9.9% (−6.7 to 22.8) between the postoperative period and one month postoperative period. Conversely, the average eGFR changes without HN were −23.9% (9.4-50.1) between the preoperative and postoperative periods and +6.5% (−1.2 to 30.7) between the postoperative period and one month postoperative period (Figure 3).

**Figure 4:**
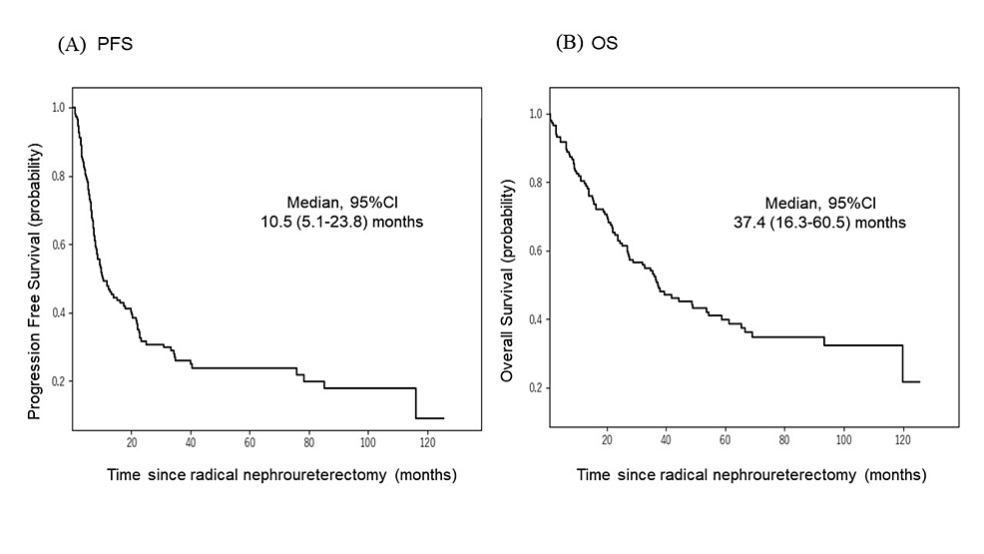
Kaplan-Meier curves of (A) progression-free survival (PFS) and (B) overall survival (OS) for all patients with UTUC. UTUC, upper tract urothelial carcinoma

**Figure 5:**
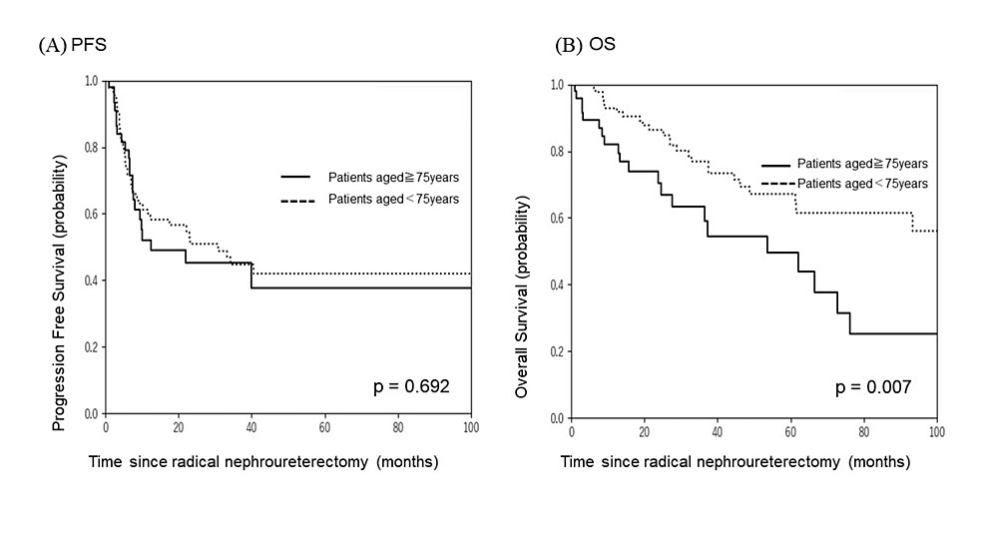
Kaplan-Meier curves of (A) progression-free survival (PFS) and (B) overall survival (OS) for patients aged <75 and ≥75 years.

The median follow-up period was 36.9 months (0.9-125.5). The median OS was 37.4 months (95% CI 5.1-23.8), and the median PFS was 10.5 months (95% CI 16.5-60.5) (Figure 4). Furthermore, the PFS showed no significant difference between nonelderly and elderly groups, and the OS was significantly shorter in the elderly than nonelderly groups (Figure 5).

## Discussion

We investigated the changes in renal function after RNU for UTUC focusing on elderly patients. Our results showed half of the elderly patients had an eGFR < 50 preoperatively and only 8% of elderly patients were cisplatin-eligible one month after RNU. Furthermore, HN was associated with a lower risk of renal function decline after RNU in the elderly group. 

In this study, 37% of the patients with UTUC had a preoperative eGFR < 50 mL/min/1.73 m^2^ and were cisplatin-ineligible [7]. These results are comparable to those of previous studies, indicating that patients with UTUC often already have preoperative renal dysfunction [5]. In all patients, the median eGFR was 14.7 mL/min/1.73 m^2^ lower from the preoperative period to the one-month postoperative period, and 23% of patients had an eGFR ≥ 50 at one month postoperatively. Previous studies demonstrated that the eGFR after RNU decreased at approximately 11.2-14.0 mL/min/1.73 m^2^ compared to that preoperatively [5,8-10]. However, these reports differ in the timing of postoperative renal function evaluation, often after three months postoperatively. Therefore, our results have standardized the timing of renal function measurement to one month postoperatively, coinciding with the discussion on postoperative adjuvant therapy based on the pathological results in real-world clinical practice. Notably, only 8% of the elderly group had an eGFR ≥ 50, making them cisplatin-eligible at one month postoperatively. Taken together with these results, we suggest that, compared to younger patients, almost all elderly patients who meet the cisplatin-ineligible at one month after RNU recommend planning perioperative treatment based on preoperative predictions of change in renal function.

Our study demonstrated the importance of not only a short-term perspective but also a long-term perspective about renal dysfunction after RNU. We found that the renal function of elderly people in postoperative five years decreased by approximately 65% compared to preoperative. Chou et al. [11] reported that during the first 1-5 years post-RNU, the mean eGFR varied between 31.69 and 35.85 mL/min/1.73 m². Conversely, Hendriks et al. [12] found no significant changes in renal function during the same period. A few studies have reported long-term changes in renal function, specifically focusing on the elderly. However, it is necessary to take into account that these studies also included cases of perioperative chemotherapy and not only the effects of surgical kidney decline. It has been pointed out that chemotherapy after RNU also causes a decrease in renal function with each increase in the number of cycles [13]. Our results suggest that proactive treatment strategies are particularly necessary for patients, especially the elderly.

The present study demonstrated that HN was associated with a lower risk of decreased renal function after RNU in elderly patients. Hashimoto et al. [9] reported that it is reasonable to speculate that the renal function of the diseased kidney is already impaired, and the decrease in eGFR after RNU is expected to be smaller in patients with HN. Moreover, Amirian et al. [14] reported that in the ureter, locally advanced tumors theoretically penetrate the muscularis propria, potentially diminishing peristaltic activity, and thus causing obstruction, with HN being the result. In another study, the renal function of patients with UTUC with HN decreased only 5.5 mL/min/1.73 m^2^ between the pre- and postoperative periods [8]. Our study showed that the eGFR of elderly patients with HN declined only to 8.3 mL/min/1.73 m^2^ between the preoperative period and the one-month postoperative period. We speculate that in particular, elderly people are thought to have a lower preoperatively healthy renal reserve capacity due to aging and other factors and are, therefore, less likely to experience a decline in renal function after surgery. In cases of preoperative HN, the possibility remains that platinum-based chemotherapy can be used as an adjuvant therapy even in elderly patients, which may be an indicator of perioperative pharmacotherapy.

UTUC is rare, and a sufficient number of cases is difficult to collect. This is why there is little evidence of established perioperative pharmacotherapy for UTUC [15]. While the subanalysis date from CheckMate 274 trials showed that adjuvant nivolumab had an insufficient effect in UTUC [16], patients with renal dysfunction were considered for adjuvant nivolumab therapy. Our study showed that, at one month after RNU, only 8% of elderly patients were cisplatin-eligible. Therefore, the opportunity of administering adjuvant nivolumab may increase in elderly patients with renal dysfunction.

Our study has several limitations. First, this was a retrospective and single-center study. Second, patients who received NAC were included, which might have affected renal function. However, the clinical outcome was comparable to those reported by other researchers, and we believe that the results were in line with real-world clinical practice. Third, long-term renal function follow-up data were lacking; however, such data were beyond the scope of our study, which focused on the one-month period following RNU when deciding on adjuvant therapy. Finally, the renal function was determined using only eGFR estimation. More detailed nephrological information is needed in the future.

## Conclusions

The change in renal function after radical nephroureterectomy is one of the most important complications because it influences an adjuvant therapy.

We suggest that patients with HN have a lower risk of decreased renal function than those without HN in elderly patients with UTUC. Moreover, we found that elderly patients with UTUC after RNU may experience a significant decrease in renal function in the long term.

In the treatment of elderly patients with UTUC, it may be necessary to estimate the transition of renal function, taking into account the presence or absence of HN, and develop an adjuvant therapy plan accordingly.
